# A Review of Recent Innovations in Remote Health Monitoring

**DOI:** 10.3390/mi14122157

**Published:** 2023-11-26

**Authors:** Ahmed Hany Dalloul, Farshad Miramirkhani, Lida Kouhalvandi

**Affiliations:** 1 Department of Electrical and Electronics Engineering, Isik University, 34980 Istanbul, Turkey; 22elec5002@isik.edu.tr; 2 Department of Electrical and Electronics Engineering, Dogus University, 34775 Istanbul, Turkey; lida.kouhalvandi@ieee.org

**Keywords:** remote health monitoring, medical services, implantable antennas, on-body wearable sensors

## Abstract

The development of remote health monitoring systems has focused on enhancing healthcare services’ efficiency and quality, particularly in chronic disease management and elderly care. These systems employ a range of sensors and wearable devices to track patients’ health status and offer real-time feedback to healthcare providers. This facilitates prompt interventions and reduces hospitalization rates. The aim of this study is to explore the latest developments in the realm of remote health monitoring systems. In this paper, we explore a wide range of domains, spanning antenna designs, small implantable antennas, on-body wearable solutions, and adaptable detection and imaging systems. Our research also delves into the methodological approaches used in monitoring systems, including the analysis of channel characteristics, advancements in wireless capsule endoscopy, and insightful investigations into sensing and imaging techniques. These advancements hold the potential to improve the accuracy and efficiency of monitoring, ultimately contributing to enhanced health outcomes for patients.

## 1. Introduction

**Figure 1:**
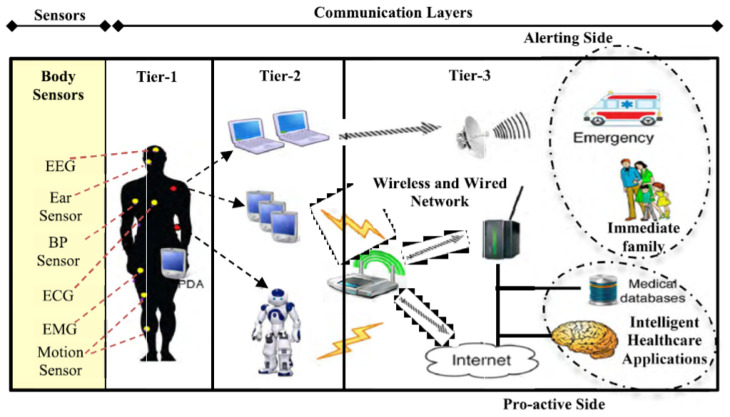
Remote health monitoring system architecture [6].

Remote health monitoring, which is also referred to as remote patient monitoring or telehealth monitoring, involves the utilization of technology to remotely monitor patients’ health conditions. This approach entails the gathering, transmission, and analysis of health data, enabling healthcare providers to monitor patients, make informed decisions, and provide timely interventions [1]. Diverse technologies and systems have been developed to facilitate remote health monitoring, including wearable devices, sensors, mobile applications, and communication networks [2,3,4]. The implementation of remote health monitoring systems has demonstrated promising outcomes in terms of improving patient results, enhancing medication adherence, and increasing patient engagement and satisfaction. It enables personalized care, facilitates remote consultations, and encourages self-management of health conditions [5]. Figure 1 provides a visual representation of the overarching structure of these systems. The primary goal here is twofold: firstly, to enhance the connectivity within the human body, thereby improving the functionality of sensing and imaging devices for early detection of conditions like tumors and glucose levels. Secondly, to optimize and advance implantable antennas and sensors utilized in a variety of devices, including wireless capsule endoscopes and magnetic resonance imaging tools. Additionally, it is worth emphasizing that Tier 1 entails the collection of patients’ vital signs through the use of interoperable wearable medical devices. These devices transmit the gathered data to Tier 2. It is crucial to note that the primary focus of this survey centers on the recent advancements in the first tier.

Remote health monitoring systems encounter numerous challenges, including issues related to data security, reliability, and power consumption [7,8,9]. Protecting sensitive medical data during transmission over wireless networks is a major concern, as it leaves the information susceptible to cyber attacks. Ensuring reliability is crucial for remote health monitoring systems, as any system failure can have severe consequences, such as delayed interventions and incorrect diagnoses. Additionally, the power consumption of wearable devices used in remote health monitoring is a critical factor, as frequent charging can be inconvenient for patients who rely on battery-operated devices. However, further research is needed to overcome these challenges and effectively utilize various technologies in healthcare to achieve optimal outcomes [10].

Furthermore, in order to investigate the safety and electromagnetic compatibility between wireless systems and medical devices, refs. [11,12,13] show the safety and electromagnetic interference within medical devices caused by wireless communication devices. Moreover, and based on international standards and reports, ref. [12] provides a recommendation which showed that two-way interference devices such as mobile phones have the highest risk whereas local network area radio has small interference results with no risk. Hence, using wireless technologies within the recommended separation distance from the medical devices stated in the standards is acceptable. On the other hand, ref. [13] experimented with electromagnetic interference coming from noisy lights within wireless medical telemetry and proposes a method to overcome this issue. Ref. [14] proposes a carbonaceous material that works as electromagnetic interference shielding to guard humans and reduce electromagnetic pollution that affects diverse wearable devices.

In this paper, we present a comprehensive overview of the most recent advancements in device development within the specific field of remote health monitoring. We also provide an overview of prior research, emphasizing their areas of focus, significance, and the primary criteria they have employed to tackle deficiencies in the current body of literature (See Table 1). This research encompasses a wide range of studies and innovations, and we have organized our discussion into two distinct sections. The first part focuses on advancements in device development [15,16,17,18,19,20,21,22,23,24,25,26,27,28,29,30,31,32,33,34,35,36,37,38,39,40,41,42] within the field of remote health monitoring. Within this section, we delve into various aspects of this technology. Refs. [15,16,17,18,19,20,21] explore different antenna designs used in patient monitoring applications, with a special emphasis on their diversity and applications. We then shift our attention to small implantable antennas for in-body critical usage and other purposes, as highlighted in [22,23,24,25,26,27,28]. On-body wearable antenna design applications are discussed in [29,30,31,32,33], shedding light on the wearable aspect of health monitoring. Lastly, we examine detection and imaging systems in [34,35,36,37,38,39,40,41,42], showcasing the versatile applications of remote health monitoring. The second part of this paper is dedicated to presenting the methods executed in these monitoring systems [43,44,45,46,47,48,49,50,51,52,53,54,55]. This section provides valuable insights into the methodology behind remote health monitoring. Channel characteristics studies for different devices are thoroughly discussed in [43,44,45,46,47,48]. In addition, advancements in wireless capsule endoscopy (WCE) are addressed in [49,50], highlighting its significance in the context of remote health monitoring. The section then concludes with [51,52,53,54,55], which focuses on sensing and imaging technique systems, providing a comprehensive understanding of the tools and techniques used in this field.

The rest of this paper is organized as follows: Section 2 provides a comprehensive overview of the recent progress in remote health monitoring devices. Section 3 presents an overview of the current methods and approaches employed in remote health monitoring. Lastly, Section 4 provides a summary of the findings and draws conclusions based on the research conducted thus far.

## 2. Advancements in Remote Health Monitoring Devices

The advancements in fifth-generation and beyond (5G/B5G) technologies are reaching a point where they can offer fast data transmission, minimal latency, and reliable connectivity. This opens vast possibilities for remote health monitoring. Recently, these technologies have been instrumental in assisting the elderly population with early diagnosis, especially when it comes to using health monitoring devices. In the preceding section, we introduced the remote health monitoring concept while pointing out some of the advantages and challenges. In this section, a comprehensive overview of the most recent advancements in device development [15,16,17,18,19,20,21,22,23,24,25,26,27,28,29,30,31,32,33,34,35,36,37,38,39,40,41,42] within this specific field is derived. These studies [15,16,17,18,19,20,21] delve more into different antenna designs used in various patient monitoring applications whereas [22,23,24,25,26,27,28] focus more on small implantable antenna for in-body critical usage and other purposes. Refs. [29,30,31,32,33] introduces on-body wearable antenna design applications and [34,35,36,37,38,39,40,41,42] targets mainly detection along with imaging systems. Table 2 provides the summary of reported studies based on remote health monitoring devices.

It should be noted that Section 2 provides detailed information on the advancements made in various types of devices, their functionalities, and the impact of these advancements on the field as a whole. It may include discussions on new technologies used, novel features incorporated, and improvements in performance, efficiency, and user-friendliness. On the other hand, Section 3 aims to present the methods executed in monitoring systems related to the field. This section focuses on describing the techniques, approaches, and procedures used in monitoring systems. This section provides insights into the methodologies utilized in monitoring systems. While Section 2 provides an overview of device development advancements, Section 3 complements it by highlighting the methods and techniques used in monitoring systems that implement these advancements. Together, these two sections provide a comprehensive understanding of the recent advancements in device development and how they are utilized in monitoring systems, enabling readers to grasp the current state of the field and the methodologies employed to monitor and analyze data.

In [15], dynamic metasurface antennas (DMAs) are used in residential sensing motion applications while utilizing radio frequency (RF) signals because they provide simplicity and an effective range. Furthermore, the proposed DMA is able to produce various reconfigurable patterns at a single frequency and detect minute movements, which is to say, small motions of human breathing in a line of sight (LoS) or non-line of sight (NLoS) manner. In addition, the DMA proposed considers single noise floor calibration and could work in different room shapes which makes it an alternative hardware solution for seamless health monitoring along with intruder detection.

**Figure 2:**
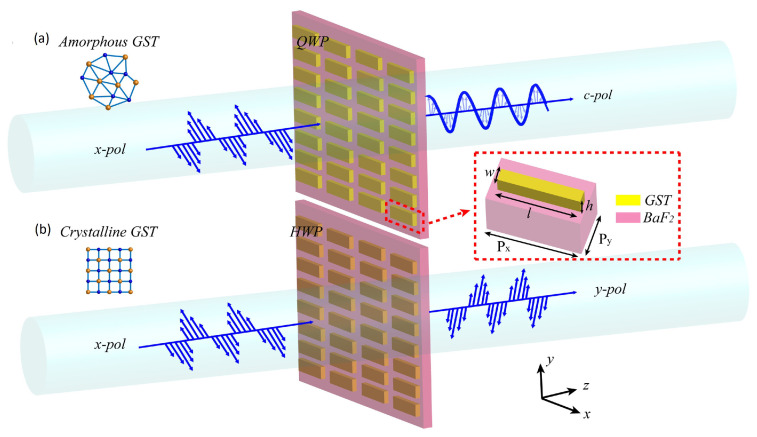
Rectangular metasurface antenna with (**a**) Quarter-wave plate in amorphous GST state and (**b**) Half-wave plate in crystalline GST state [16].

Moreover, ref. [16] presents another metasurface antenna design that can be utilized in optical sensing for remote health monitoring systems. Ref. [16] is based on an active rectangular wave plate antenna metasurface that uses phase-changing $Ge_{2}Sb_{2}Te_{5}$ (GST) material which has a high conversion rate along with high transmittance efficiency. The original metasurface works at a wavelength of 10.0–11.9 μm with a quarter-wave plate; however, using GST in the state of crystal-line, the metasurface wavelength is 10.3–10.9 μm with a half-wave plate. Figure 2 demonstrates the two-wave plate metasurface. The utilization of the electric and magnetic resonance dipoles results in higher efficiency related to the transmittance along with the polarization conversion rate (PCR) of 99.9%.

Furthermore, ref. [17] presents another antenna design where spoof surface plasmon polaritons (SSPs) are used with a groove metal antenna that is excited via a coupler of 1D dielectric gradient metasurface (GMS). Also, the performance of the sensing in the THz region was investigated in the design. The proposed design of the SSP does not face the problems of metal counterparts that have an antenna with sub-wavelength-like cheap efficiency together with absorption loss. This structure permits a design of compact along with ultrathin sensing devices that could be used in wireless monitoring of health implementations. The results show that the SSP GMS with 0.46 THz of resonant frequency exhibits 517.9 GHz/RIU of high sensitivity with a 0.0001 RIU resolution and a 262 of high Q-factor is obtained. Also, an investigation on 410 GHz/RIU sensitivity with a 0.05 RIU resolution could be achieved to predict the changes in the refractive index.

**Figure 3:**
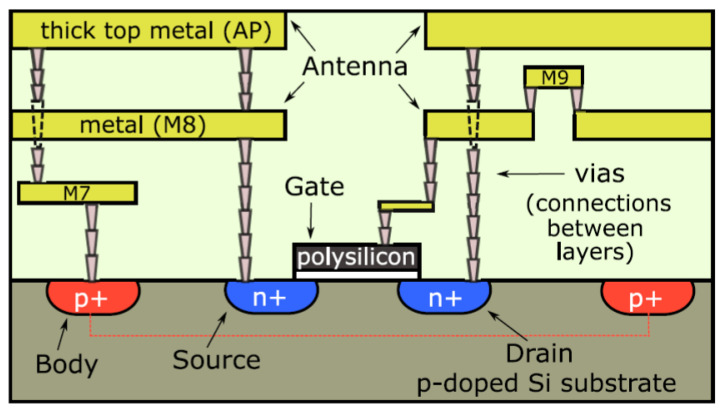
The chip with the proposed log-spiral antenna [18].

Moreover, ref. [18] uses a different antenna technique. Ref. [18] introduces a method of passive imaging using radiation emitted by the human body in the lower THz frequency range. A broadband uncooled detector is utilized for this purpose. The detector comprises a Si CMOS field-effect transistor integrated with a log-spiral THz antenna shown in Figure 3. Experimental measurements indicate that it exhibits a relatively consistent responsivity across the frequency range of 0.1 to 1.5 THz. The optical responsivity of 40 mA/W and 42 pW/$\sqrt{Hz}$ noise-equivalent power of the sensor are obtained. These measurements align well with simulations, which suggest an even wider responsivity range beyond 2.0 THz. Also, through the process of capturing an image of the fingers of a hand, which has dimensions of 2.3 × 7.5 cm${}^{2}$, using a pixel size of 1 mm${}^{2}$ and scanning at a speed of 1 mm/s, impressive results are obtained. The achieved SNR ratio is 2, indicating the quality of the captured image, and the noise-equivalent temperature difference is 4.4. Hence, this design is capable of being integrated into remote health monitoring for THz frequency detection applications.

Further wideband spiral antenna type topology in [19] for ingestible capsule endoscope systems is proposed where it is employed for imaging and detecting applications in wireless health monitoring. Also, an experiment comparison for the utilization of the design in a human and anesthesia pig is demonstrated. The wireless capsule system uses a tiny antenna to send real-time images at a high resolution to the receiver using the on–off keying (OOK) modulation technique. To send the images without worrying about the capsule’s position, the antenna inside the capsule uses an isotropic radiation pattern. Furthermore, to verify the antenna performance design, the return loss and received power were measured by a circularly polarized antenna used in an anesthesia pig and the result demonstrated the same performance as the antenna capsule used in the human phantom.

Furthermore in [20], a symmetrical UWB dual-loop antenna design that works in the ISM band is demonstrated. This design can be used in various biomedical applications such as wireless ingestible endoscopy. In addition, the antenna consists of two symmetrical loops in the center with feeding and parasitic patches. The simulated result showed that the UWB of 143% ranging from 1.11–6.03 GHz could be obtained in the ISM band by utilizing two rectangular patches together with putting the feeding direction to $90^{\circ }$. The designed antenna is also demonstrated in pork and the result showed a bandwidth of 124%.

In [21], an antenna-in-package design is introduced for a wireless ingestible capsule that works at the ISM band. The design aims to send the data between the capsule and the Bluetooth technology using a smartphone. The antenna structure uses a modified inverted-F structure to achieve size reduction and polarization diversity. Thereafter, a comprehensive evaluation method is used to analyze the performance of radiation. The experiment result shows that the given design could achieve good margins for the link regardless of the capsule orientation. Moreover, the antenna is not sensitive to digestive organs and the reflection measurement can be obtained using a muscle-mimicking phantom.

In addition, the work in [22] designed a capacitance-loaded wideband implantable antenna employed for WCE. In this design, a carve on the radiator and the ground plane of the antenna is considered to reduce the size and increase the bandwidth impedance. Furthermore, a capacitance loading method is utilized to enhance the impedance matching. Moreover, an experiment applied on a chicken breast shows that the performance of this design outperforms other designs in terms of impedance bandwidth, radiation pattern, and certain absorption rate. The simulation results of the impedance BW were 2.17–2.69 GHz 20.5% for $\left|S_{11}\right|$ less than −10 dB and with a drastically smaller volume of 120 mm${}^{3}$.

In [23], a study uses a 434 MHz low-profile ultraminiature wireless in-body antenna for implantable and ingestible usage in telehealth monitoring. The proposed system uses 50 Ω input impedance whereas the antenna utilizes an analytical approach to miniaturize and protect it from detuning the impedance. In addition, an enhancement in the inner surface of a biocompatible capsule is done as well. The proposed antenna is investigated with a high-permittivity capsule in heterogeneous phantoms that matches the gastrointestinal tract tissue to improve its robustness. The measurement results demonstrate an agreement in terms of the performance of the radiation along with reflection coefficients.

In [24], a circularly polarized antenna is proposed for wireless implantable applications in remote patient monitoring at the ISM band. The input impedance of the antenna along with minimized ground in lossy muscle tissue are studied. The antenna employs LC element plus arc-shaped slots together with shorting pins to increase the impedance matching. A compacted antenna size of $\pi x4.8^{2}$ was obtained and the simulation demonstrates good impedance matching together with axial ratio and a circular polarization bandwidth of 18.3%.

The authors of [25] introduced a topology that uses a linear polarized, highly directed on-chip dipole antenna in a 60 GHz mm-wave radio. The system uses 1.5 T MRI and follows time division multiplexing (TDM) with the OOK modulation technique to minimize the multiple wireless link channel interference. As a result of the on-chip dipole antennas, the raw BER is $10^{-6}$ for a 10 cm that overcomes the raw BER of WiFi which has a $10^{-2}$ 802.11n system or a Bluetooth system with $10^{-3}$. Hence, this topology can contribute to the wearable wireless MRI receiver in remote health monitoring applications.

**Figure 4:**
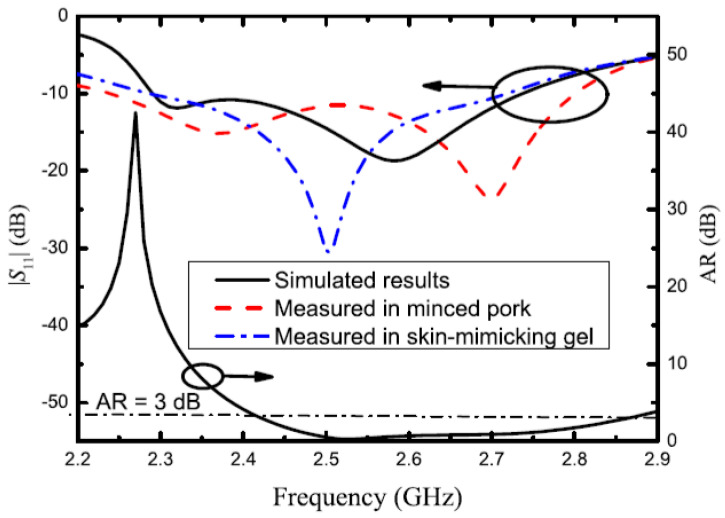
Measured and simulated results using circular polarization antenna in different tissues [26].

The work in [26] proposes an implantable antenna design that uses circularly polarized (CP) antenna at the industrial, scientific, and medical (ISM) band for diagnosing and physiological parameters monitoring in remote health monitoring systems. The antenna design contains a pin-loaded patch that has a pair of two non-degenerated orthogonal modes together with two open-ended slots. In addition, a via walls around the pin-loaded patch is employed within this design to protect the proposed antenna from loss of gain in lossy tissues. Furthermore, the simulation demonstrates an impedance of 23.1% with 12.8% bandwidth of the CP antenna and a 1.5 dBi enhancement in the antenna gain. Also, the studied antenna was manufactured and tested. The result of this examination meets the simulation result as it is demonstrated in Figure 4.

**Figure 5:**
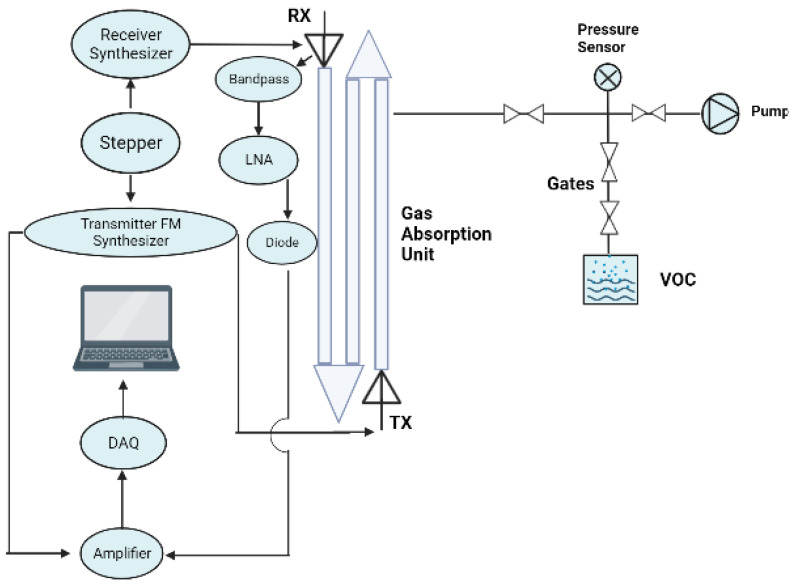
Transmitter and receiver gas spectroscopy sensors diagram integrated with absorption cell.

Furthermore, the authors in [27] review an outcome of a previously designed proposal based on a sensor system used for gas spectroscopy and telehealth diagnostics of human breath. The system contains a transmitter along with a receiver manufactured using IHP’s (0.13 μm SiGe BiCMOS) technology. A usage of a mm-wave/THz system that embraces the length cell of a folded gas absorption with integrated antennas at 238–252 GHz and 494–500 GHz while employing a fractional integer-N phase-locked loops (PLLs). Figure 5 shows the general structure of the proposed sensor technique. The updated system is more compact compared to the previously designed system and has 52 s time acquisition compared to 133 s in the previous system.

Moreover, the dual-band implantable antenna designed in [28] employs a three-layer modal of effective relative permittivity ($\varepsilon_{eff}$) among human lossy tissue superstrate. The antenna can work at both wireless medical telemetry service (WMTS) 1.4 GHz and ISM band applications. In order to obtain a CP on the design, two arc-shaped tails with open ends were employed in the design. The experiment demonstrates a CP bandwidth of 10.38% in the WMTS band along with an impedance bandwidth of 21.3% in the ISM band.

In [29], a design on the indoor human body 3D limbs motion detection utilizing impulse radio-ultra wideband compact and cost-effective on-body antennas is discussed. The body-centric wireless channel characteristics were investigated with various parameters such as the magnitude of the path loss, number of multipath components, RMS delay spread, and Kurtosis while considering the LoS and NLoS paths. Furthermore, the accuracy of the signal at the receiver is found to investigate the pulse-preserving nature of the wearable antenna and the result manifests high accuracy localization of 90% in the 0.5–2.5 cm range. Hence, this method of wireless body sensor network contributes to healthcare and patient monitoring applications.

In addition, the authors in [30] studied the realization of conformal wearable transparent antennas that can be employed in real-time body-centric communication applications like wireless health monitoring for older people and dementia care people. This design employs a mesh conductive sheet along with polydimethylsiloxane (PDMS) polymer as the substrate. This technique is simpler/cost-effective and the antenna is also much more flexible/robust in bending. Moreover, a prototyped dual-band antenna works in ISM along with the wireless local area network (WLAN) (2.33–2.53 GHz and 4.7–5.6 GHz) which has been introduced to verify the design. In order to improve the gain together with the efficiency, the RF performance was enhanced via the utilization of a two-layer conductor.

In [31], a human body-worn antenna is utilized with 12 sensors while considering different base stations, time of arrival (ToA), and first peak detection techniques in indoor UWB for human motion detection that can be used in remote health monitoring. To find the absolute displacement error, a 2D localization error was obtained with 8-TX, and the accuracy was calculated in an optical motion system. The resulting error was by a third better compared to the commercial optical system. Moreover, the cuboid-shape configuration with 4-TX results in an average percentage error of 2–3% (0.5–1 cm reduction in accuracy) than for the Y-shape 4% (1–1.5 cm reduction in accuracy), and yet the Y-shape is further compacted and simple which allows it to be used in healthcare monitoring.

The work in [32] investigates the on-body triband coil antenna that uses an impulse radio (IR) wireless capsule endoscope (WCE) system for remote patient monitoring. The designed antenna uses three operating frequencies. At first, a discussion that utilizes a low-frequency multi-band communications method to transmit images is discussed. Furthermore, to test the transmission performance, three in-body transmitting antennas along with different parameters are designed and simulated. The results demonstrated that 50 mm attenuation has 32, 43, and 52 dB at the three operating frequencies which is 10.3, 13.3, and 16.4 dB attenuation growth compared to simulated results.

**Figure 6:**
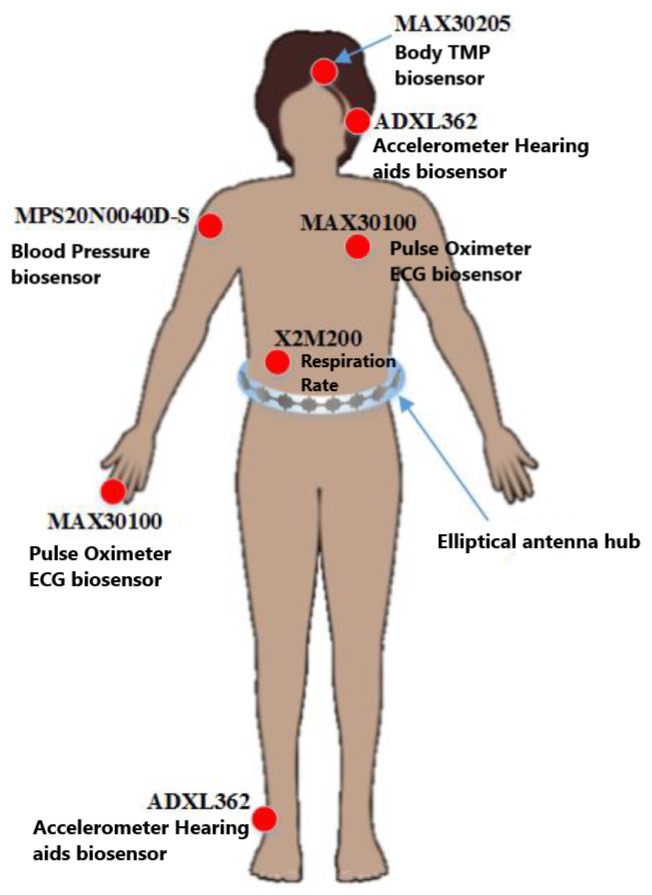
IoMT-based WBAN elliptical antenna system and sensors scheme [33].

The work in [33] presents a new worn antenna technique that uses the Internet of Medical Things-based wireless body area network (IoMT-based WBAN) for health monitoring applications. In order to obtain simultaneous orthogonal signals in the connection and immunity from possible interruptions, the sensors will use Walsh-Hadamard coding along with an elliptical leaky-wave antenna (LWA) placed on the hubs. Figure 6 presents the proposed sensors together with the elliptical hub antenna. Furthermore, the results of measurements and simulations stated that the on/off body connection was enhanced compared to formal antenna design and agrees with the results of the calculations. Both results had radiation pattern ripples about 3 and 3.5 dBi in the X-Z plane along with $68^{\circ }$ and $72^{\circ }$ half power beamwidth at the Y-Z coordinates.

**Figure 7:**
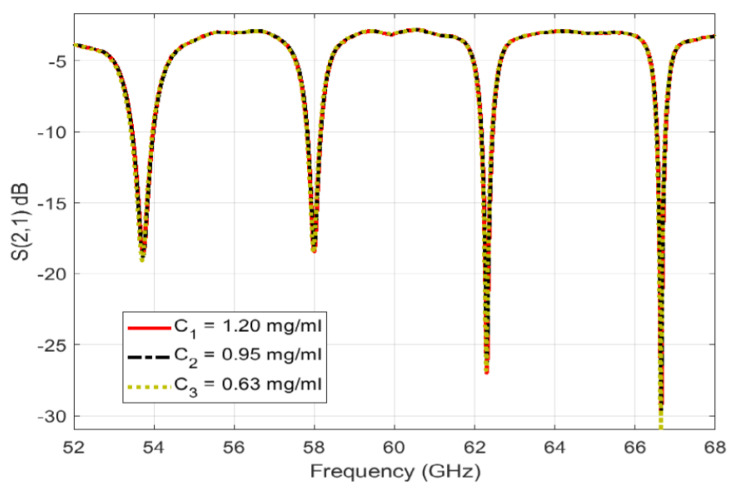
Measured results of WGM resonance sensing employed with different glucose-tested concentrations [34].

**Figure 8:**
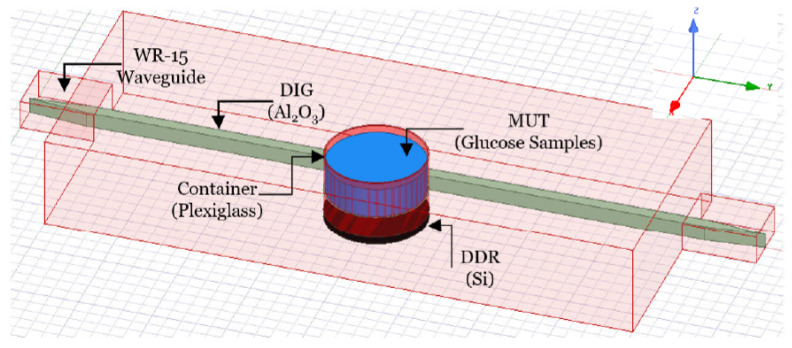
The structure of the wireless WGM sensor [34].

On the other hand, ref. [34] proposes a commercial coaxial probe kit utilized in water-glucose samples. This design aims to obtain the sensitivity in low variation frequencies of the glucose concentrations along with the change in the permittivity of the dielectric and tangent loss. Figure 7 shows the results with three concentrations. The relative permittivity of water-glucose in the mm-wave range of 50–67 GHz is utilized by the Whispering Gallery Modes (WGMs) bio-sensor sensing system. Figure 8 presents the WGM sensor design. This sensing structure detects the magnitude and phase variations of WGM for glucose continuous monitoring which could be used for telehealth applications. The results show that the design has a (2.5–7.7 dB/[mg/mL]) performance of sensitivity.

The authors in [35] present a hybrid breast cancer detection technique that contains thermography and high-frequency excitation methods at the same time. This method utilizes the distribution as well as the variation of the breast temperature in order to predict the position along with the size of the cancerous tumor. Moreover, the heat transfer equation is also utilized to find the surface temperature distribution and the results demonstrate a direct relationship between both temperature/specific absorption rate and tumor position together with the size. Thus, this breast cancer detection method could be implemented in wearable devices for remote patient purposes.

**Figure 9:**
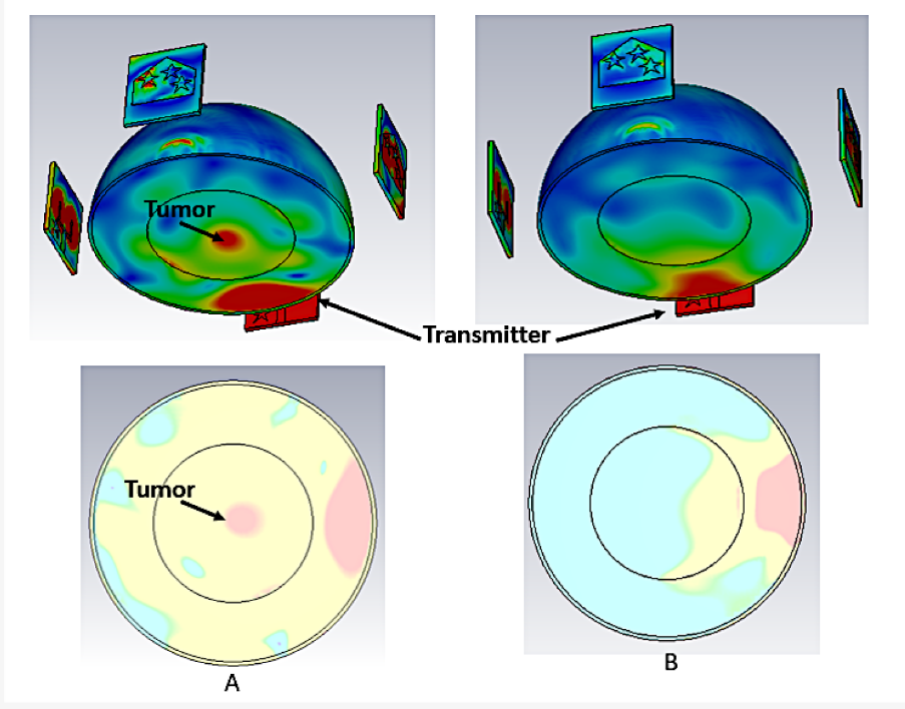
Tumor detection simulation results: (**A**) unhealthy breast, (**B**) healthy breast [36].

Further UWB antenna design is introduced in [36] for breast cancer detection applications. The design consists of a tapered slot ground, a rectangular slotted patch, and four star-shaped parasitic components. The proposed antenna system offers a realized gain of 6 dBi and an efficiency of 80% on the radiation bandwidth. Furthermore, the antenna exhibits excellent directionality and minimal signal distortion. In addition to that and to validate its performance, a breast phantom was constructed, and an antenna array was positioned over the breast to collect the reflected and transmitted waves for tumor characterization. Figure 9 presents tumorous and healthy breast detection results.

The work in [37] provides a biosensor design employing a split-ring antenna resonator for glucose and cancer detection. The frequencies of the detection mechanism depend on the properties of the structure like electrical permittivity. Furthermore, the glucose oxidase enzyme was merged to result in biospecificity for glucose. The result of the red shift in the frequency demonstrates that the response for DI water and glucose verifies the theoretical expectations with the highest error rate of 7.3% and has a frequency shift of 17.5 MHz in 15 min along with the sensitivity of 0.107 MHz/mg mL${}^{-1}$.

The authors in [38] introduce a metamaterial antenna array design used for imaging and detecting breast tumors in biological tissues. The designed transceiver antennas contain square concentric rings connected to a central patch placed around a breast model. Furthermore, the result shows an average radiation gain of 11 dBi and efficiency enhancement of 18% compared to the conventional patch antenna array. Also, an average reflection coefficient was obtained better than S11 ≤−20 dB which means it detects weak signals as well as reduces the distortion and has a high average isolation of 30 dB between the radiators.

In [39], a 2.4 GHz continuous wave (CW) coupled with a 2D nine-element antenna array radar system that uses a signal processing technique is presented for remote monitoring of the human respiratory rate system. Measurement results indicate that the proposed design is capable of imaging the respiratory system within the body and obtaining a precise respiration rate for it. Moreover, the design accuracy was investigated by recording the ribcage circumference while utilizing a piezoelectric respiratory sensor. The result obtained a 0.05 m average imaging error with a 30 ms error in estimating the respiration intervals.

Another imaging technique is presented in [40]. This considers a photoconductive antenna/photomixer array element as a continuous-wave terahertz with its ray characteristics. The design gives the advantage of increasing the terahertz power with less power consumption. Small μW terahertz power could be found using the array structure at 1 THz. In addition, radiated power reliance on the beat frequency together with the applied DC bias has been shown. It was demonstrated that the radiated ray could be directed more than 30${}^{\circ }$ by modulating the angle between the two exciting laser rays. Hence, the design could be used in monitoring and medical imaging applications.

Upcoming study [41] presents the SPR relation with the fluorescence of the dye molecule. The primary goal is to harness the plasmon hybridization modes of nanoparticles in order to achieve the maximum enhancement of nearfield augmentation. This enhancement is crucial for enhancing both the lifetime and quantum efficiency of dyes used in deep-tissue imaging for telehealth monitoring applications. An investigation of nanoparticles (NPs) with strong plasmon resonance is provided in order to extend the visible near-infrared (NIR) spectra. The fluorescent improvement in dye molecules that are presented as a function contains the distance of NP’s surface, NP’s radius, and the thickness in the inner and outer core/shell was delivered.

In [42], a wireless non-battery trimodal neural interface system-on-chip (SoC) that uses eight electrical channel stimulation with two sixteen channels for both neural recording and optical simulation is proposed. The design experimented in vivo and in vitro while using four anesthetized rats to test the trimodal SoC system and the result illustrated the neural activities and immunostained tissue responses that can be employed in various wireless patient monitoring utilization.

## 3. Current Methods and Approaches in Remote Health Monitoring

In remote health monitoring systems, the type of devices and the employed approaches concurrently play important roles. In the preceding section, the various devices used in health monitoring are described in detail. This section is devoted to presenting the methods executed in these monitoring systems [43,44,45,46,47,48,49,50,51,52,53,54,55]. Specifically, refs. [43,44,45,46,47,48] present channel characteristics studies for different mentioned devices whereas [49,50] are devoted to WCE advancements. Refs. [51,52,53,54,55] address sensing and imaging technique systems. Table 3 describes an outline regarding the described literature in this section.

In [43], an empirical channel model in human body communication (HBC) that uses impulse response is studied. The modeling of the channel variations in a wide range (5–80 MHz) could be found by obtaining the magnitude and the phase of the impulse response presented by a series of random variables measured using the optical-synchronization method of 70 human subjects. Furthermore, each user of the HBC has a different signal loss due to the dielectric material difference. The normality verification test demonstrated that the random variables followed a normal/uniform distribution with a mean and standard deviation. Moreover, the result showed a dependency on the impulse responses at each sampling point. Hence, the design is capable of reliable data communication in medical health applications.

In [44], a study is presented on data rates, resolution, and the transmission power for an in-body to on-body ultra-wideband communication (UWB) channel used for early-stage telehealth disease detection in WCE. The power is studied in three cases: Channel capacity, capacity with pulse-position modulation (PPM), quadrature phase shift keying (QPSK) modulation, and PPM with QPSK modulation while employing Reed-Solomon (RS) channel coding. The result demonstrates power saving while operating in the minimal frequency ranges whereas capacity and the power transmitted remain the same even when utilizing a bandwidth bigger than 1 GHz. Moreover, the performance of PPM and QPSK do not differ much.

In addition to the previous study, [45] shows further a dual analyte channel with a highly sensitive photonic crystal fiber (PCF) bio-sensor using surface plasmon resonance (SPR). The designed sensor is characterized via a finite element method (FEM). The proposed design shows a result of 186,000 nm/RIU for wavelength sensitivity (WS) and amplitude sensitivity (AS) of 2792.97 ${\mathrm{RIU}}^{-1}$. Thus, the proposed bio-sensor could be used in detection assessments such as bio-molecules and biological analytes for health monitoring applications.

The work in [46] presents interbody channel characteristics for Invivo wireless nanosensor networks at both terahertz bands 0.1–10 THz and optical frequencies 400–750 THz. This study could contribute to different applications such as intrabody patient health monitoring and accurate disease diagnosis. The path loss was obtained by considering the large and small scales scattering particles, spreading propagating wave effect, and human tissue molecular absorption. The analytical results were tested using EM simulation and the results validated each other. Furthermore, the design investigates the concept of links budget in human body nanodevices where the power of the transmitter, path loss of the medium, and the sensitivity of the receiver were considered in THz and photonic devices.

Furthermore, the work in [47] studies the propagation of a wave close to the human body surface that uses a body antenna network (BAN) for power-efficient on-body propagation wireless medical sensors. The mechanism between the sensors utilized is the Norton wave to deal with the band-aid antennas. In addition, the Norton wave frequency at 3 GHz demonstrates that the thinner outer surface layers are not important and the two-layer structure model is precise enough to find the path gain. The path gain will be frequency dependent with small ranges; however, when using UWB the Norton surface channel will be dispersionless which is an advantage in terms of communications. Furthermore, a comparison between the proposed design and other published experiments is investigated.

The authors in [48] propose a performance analysis using an integrating dual data rate receiver that allows broadband human body communication to operate with CW, amplitude-modulated (AM), and frequency-modulated (FM). This method improves the security and energy efficiency of WBAN monitoring applications. Furthermore, a numerical solution is provided with simulations that investigate the receiver with 22 dB efficiency in SIR for both AM and FM. Moreover, measurements of the proposed design were applied to signals passing from the human body and obtained using an oscilloscope. This measurement shows a 10${}^{-4}$ BER for CW, AM, and FM with −21 dB efficiency in interference rejection.

In [49], a design of backscatter RF is introduced in wireless high data-rate communications with deep medical implants. This design is power efficient since it allows for remote information reading and eliminates about 20–45 mW by removing the transmitter at the implant. The structure designs a self-resonant reconfigurable antenna system in WCE that produces a large radar cross-section. Furthermore, bistatic on-body antennas are proposed for the sake of reading the information from the implant and to enhance the backscatter connection performance. The proposed design was verified using numerical computations along with an experiment in liquid phantom and in-vivo animals. It was observed that the capsule has 1 and 5 Mb/s backscatter data connectivity at a depth of 13 cm with a 250 mW moderate reader power.

In addition, [50] investigates the localization of a WCE system through the gastrointestinal (GI) tract for remote patient applications. Furthermore, a 3D full-wave simulation of the human body was employed to find the bounds in the stomach and the tiny and large intestines. The study considered various parameters that affect localization accuracy such as array sensor order, organ properties, and number of pills. It also studied the case when an arbitrary transmitting signal follows a determined probability distribution. The result of the computational solver demonstrates that the external antenna receiver number has a higher effect on localization precision compared to the number of capsules used in the GI. Also, it shows that the large intestine is impacted most by the power of transmitter probability distribution.

**Figure 10:**
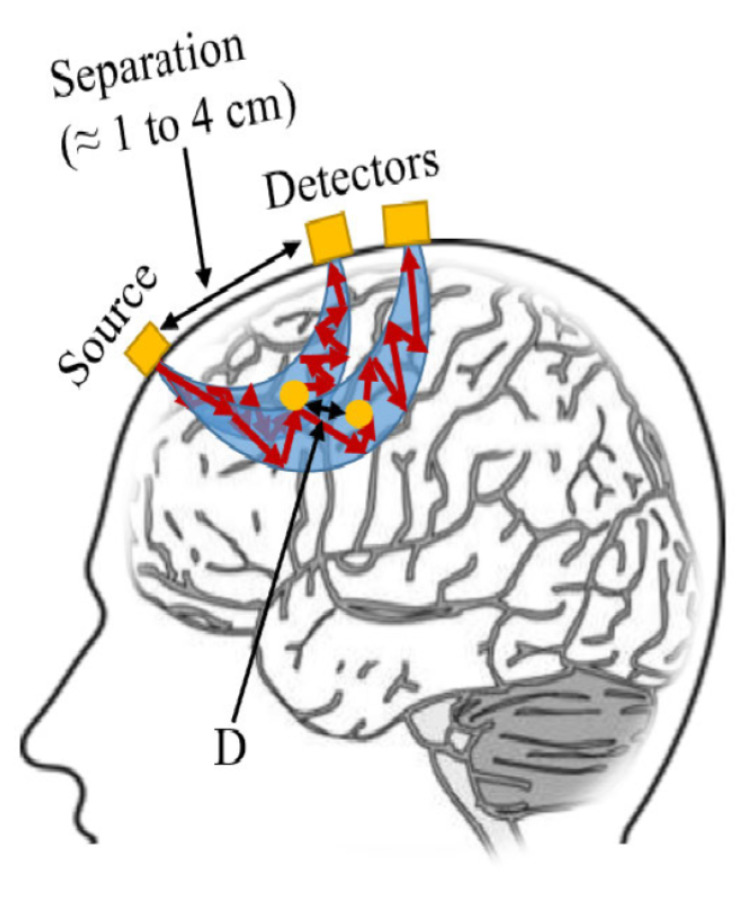
An illustration for the method of finding certain brain areas within a specific distance to enhance optical brain imaging [51].

The authors in [51] review various designs used for improving the spatial resolution of optical brain imaging (OBI) systems. OBI systems have features in terms of low cost, portability, and ease of usage compared to other biomedical imaging systems; however, they lack spatial resolution, especially for brain image applications. Moreover, it has two primary bottlenecks which are the low penetration of the light NIR over deep tissues and the unknown path of the light NIR photon. This overview considers the principles of OBI systems with their challenges in spatial resolution improvement. Moreover, it summarizes different methods to solve the spatial resolution problem and compares them with better spatial resolution instruments. Figure 10 presents one of the methods to enhance spatial resolution by measuring two areas within the brain.

The authors in [52] present an overview in terms of functions, methods of printing, performance, and the material of physical sensing devices along with their interface circuits for wearable health-monitoring applications. These sensor devices are employed for the sake of monitoring various parameters of biometrics like skin temperature, human pulse wave pressure, and human motion strain. Moreover, the recent progress of the printed interface circuits integrated with wearable sensors is investigated as well while looking at their structures along with their function. These are interface circuits such as interconnects, thin-film transistors, digital circuits, amplifiers, and antennas.

Further study in [53] investigates the disturbed transmitted and received signals caused by complex EM waves that result in diffraction along with wave surface propagation which is presented. This design considers two antennas to attain the transmission changes in the mm-wave glucose sensing system. Furthermore, an experiment that includes glucose together with numerical simulations is held and it showed that the received signals are dominated by the unwanted contribution—multipath transmission—however, it is minimized via employing absorbers around the skin tissue model that contains the sensors of the antennas. Hence, the signal sensitivity of the proposed system using glucose was enhanced (change in transmission $\Delta S_{21}$).

In this novel study [54], an investigation on near-field THz imaging using various biological samples is introduced to overcome the spatial resolution in imaging applications. The design uses probes made from metal, quartz, and telfon integrated with a horn antenna. Furthermore, those probes were employed to image a mouse brain, a dwarf umbrella tree leaflet, and a bug wing. The results of imaging a mouse brain showed different regions from certain sections of the brain; however, in the leaflet, the image of venation was obtained. Moreover, the bug wing imaging demonstrates a different transition from corium to membrane regions and it was shown that it has a different profile compared to the optical image. Further implementation of the design using a dielectric slab with an inside structure for imaging was introduced as well.

In [55], an ultrashort pulse microwave-induced thermoacoustic imaging technique is integrated with a large aperture antenna to overcome the large field imaging problems. This design has a 40 cm × 27 cm microwave radiation, 14 cm × 14 cm uniform imaging view, a 7 cm depth of imaging, and a resolution of 290 μm. Moreover, the designed system was examined with breast tumors in several shapes of phantoms and while using ex vivo human breast tumors with ewe excited breast (π × 5 cm × 5 cm) where the tumor has a 1:2 contrast rate. The results demonstrate that the system can precisely allocate breast tumors and has potential in breast screening applications.

## 4. Conclusions

This paper has provided a survey of recent advancements in the development of remote health monitoring devices. The scope of this research spans a range of domains, covering antenna designs, small implantable antennas, on-body wearable solutions, and versatile detection and imaging systems. Furthermore, we have undertaken an in-depth exploration of the methodological approaches applied in monitoring systems, encompassing channel characteristics studies, advancements in wireless capsule endoscopy, and insightful investigations into sensing and imaging techniques. Despite these remarkable advancements, challenges such as data privacy and security, regulatory frameworks, and disparities in access to technology remain. Addressing these concerns will be crucial for the widespread adoption and equitable implementation of remote health monitoring devices. In addition, the continued development and integration of remote health monitoring devices holds great promise for the future of healthcare, offering new opportunities for preventive care, early intervention, and personalized treatment plans.

## Figures and Tables

**Table 1:** Summary of recently published surveys related to remote health monitoring.

Ref.	Year	Content Description of Each Survey
[56]	2015	Reviewed three healthcare algorithm applications using wearable inertial sensors
[57]	2016	Surveyed different encryption schemes to assure data privacy for medical cyber-physical systems
[58]	2016	Overview of current remote patient monitoring systems utilized to find multiple physiological parameters
[59]	2017	Overview of remote health monitoring applications in smartwatches
[60]	2018	Provided low bandwidth and power sensor design for remote health monitoring systems
[61]	2018	Proposed four-tier security architecture and reviewed various research works to enhance the wireless body area networks’ security and privacy
[62]	2018	Summarized health monitoring systems in health smart homes for dependent and elderly people
[63]	2019	Proposed and surveyed diverse technologies of smart health monitoring
[64]	2021	Reviewed the global navigation satellite system bridge structural health monitoring applications
[65]	2021	Provided remote health monitoring application for clinical workload decreasing during the COVID pandemic
[66]	2022	Investigated and overviewed multitemporal interferometric synthetic aperture radar used in infrastructure health monitoring
[67]	2022	Investigated the benefit of using continuous remote patient monitoring on coronavirus disease 2019 (COVID-19) patients to decrease returns to the emergency department
[68]	2023	Survey on IoT remote health monitoring security using different techniques
[69]	2023	Overview of different resource-constrained systematic remote pedestrian localization systems
[70]	2023	Overview of research works on pandemic patients’ remote health monitoring systems
[71]	2023	Reviewed recent reinforcement learning techniques for intelligent healthcare systems
**This survey**	2023	Provides a detailed overview of different antenna designs with their adaptable uses in patient monitoring, compact in-body implantable antennas, wearable antenna designs for on-body applications, detection and imaging systems, channel property investigations across various devices, advancements in wireless capsule endoscopy (WCE), as well as sensor and imaging technique systems.

**Table 2:** A Synopsis of Research on Remote Health Monitoring Devices.

Ref.	Scope	Contribution	Specifications
		**Antenna design**	
[15]	Motion detection sensing utilizing dynamic metasurface antennas.	Serves several KHz with a simple structure.	High fidelity in different room shapes. Ability to detect temporal signatures.
[16]	Implements a GST material with an active rectangular wave plate antenna metasurface for optical sensing applications.	Provides a high polarization conversion rate and high transmission efficiency.	Meets 9.3–10.7 μm wavelength while using half wave plate GST with 99.9% polarization conversion rate.
[17]	Groove metal antenna excited by dielectric gradient metasurface with spoof surface plasmon polaritons for THz sensing.	Overcomes the problem of efficiency and absorption loss to design compact and ultrathin sensing devices.	Meets 517.9 GHz/RIU of high sensitivity at 0.46 THz with 0.0001 RIU resolution and a 262 high Q-factor.
[18]	Presents THz human body passive imaging technique	Design compact detector using THz log-spiral antenna	40 mA/W optical responsivity with noise-equivalent power of 42 pW/$\sqrt{\mathrm{Hz}}$, a finger picture with 2.3 × 7.5 cm${}^{2}$ size and 1 mm/s speed.
[19]	Provides a spiral wideband antenna for capsule endoscope systems.	A design of a tiny antenna that sends high-resolution images.	A 2 voltage standing-wave ratio, a relative permittivity of 56.91, and a conductivity of 0.97 S/m.
[20]	Implements a dual-loop antenna for UWB wireless endoscopy design.	Works in the ISM bands with valid bandwidth and has gained advantages over other antennas.	A feeding direction of $90^{\circ }$ with demonstrated bandwidth of 124%.
[21]	Provides in-package antenna design for ingestible capsules.	Works at the ISM band and transmits images from the capsule to a smartphone using Bluetooth.	A good link is obtained needless of the capsule orientation and the antenna SAR meets IEEE standards.
		**Implantable Antenna**	
[22]	Provides a design of wireless-capsule endoscope antenna.	Using capacitance loading to improve the bandwidth with the impedance matching and to reduce the size of the antenna.	Meets bandwidth of 2.17–2.69 GHz 20.5% for $\left|S_{11}\right|$ less than −10 dB with a small volume of 120 mm${}^{3}$.
[23]	Low-profile ultraminiature antenna for implantable and ingestible applications.	Protect the antenna from EM properties, high-permittivity capsule inner surface improvement, and enhanced robustness.	434 MHz ultraminiature antenna with 17 mm inner surface, 50 μm substrate thickness, and −19.6 dBi gain.
[24]	Implements a circularly polarized antenna for biomedical applications.	Two orthogonal modes are obtained to enhance the antenna impedance matching.	Meets a compact antenna design that works in the ISM with a circularly polarized bandwidth of 18.3%.
[25]	Establish a wireless link for 1.5 MRI to reobtain the image.	Creates multiple links with low channel interference.	A raw BER of $10^{-6}$ which overcomes the raw BER of WiFi which has $10^{-2}$.
[26]	Proposes implantable circularly polarized antenna for diagnosing and monitoring applications.	Provides gain improvement for the antenna.	Meets 1.5 dBi antenna gain improvement, a bandwidth of 12.8%, and an impedance of 23.1%.
[27]	Provides gas spectroscopy sensor at mm-wave and THz regimes.	Implements a more compact design with less time acquisition.	The technology with the mm-wave and THz regimes has a time for 52 s acquisition.
		**Wearable antenna**	
[28]	Presents dual-band implantable antenna.	Determines the relative permittivity of the antenna using a three layers model.	Meets 10.38% bandwidth in WMTS and 21.3% impedance BW of 21.3% in ISM band with a maximum gain of −32, −31.6 dBi at the 1.4, 2.45 GHz.
[29]	Provides radio UWB antenna to obtain indoor human body and limb movements.	Uses cost-effective and simple design for wireless healthcare applications.	Investigates the channel with 90% accuracy localization.
[30]	Provides conformal antenna design using mesh conductive polymer.	Excellent performance for the human body, cost-effective, simple, and flexible in bending.	A two-layer conductor that improves the gain and efficiency at the wireless local area network and ISM bands.
[31]	Ultra wideband wearable antenna to detect human motions.	12 in-body antenna body that uses UWB technology for indoor detection.	Meets 2–3% average error compared to 4% for Y-shape.
[32]	Implements an impulse radio triband coil antenna for wireless capsule endoscope.	Image transmitting using low-frequency multi-band communications.	50 mm attenuation has 32, 43, and 52 dB at three operating frequencies with 10.3, 13.3, and 16.4 dB attenuation growth compared to simulation.
[33]	Present IoMT-based WBAN for health monitoring application.	The on/off body link improved	A 5.8 GHz LWA frequency with radiation pattern about 3 and 3.5 dBi $68^{\circ }$ and $72^{\circ }$ half power beamwidth.
		**Detection and Imaging**	
[34]	Coaxial probe kit, whispering gallery modes, and dielectric disc resonator for glucose-water monitoring at mm-waves.	Observes the sensitivity in low variations frequencies, permittivity change in the permittivity, and tangent loss.	Meets a performance sensitivity of (2.5–7.7 dB/[mg/mL]) at the lower order of WGH${}_{600}$ and WGH${}_{700}$.
[35]	Hybrid breast cancer detection by thermography and high-frequency excitation technique.	Locates the cancer tumor with size prediction.	Specific absorption rate with the location and size of the tumor.
[36]	Implements an antenna for UWB imaging applications	Enhancment in both gain and BW of the antenna	91.6% direction of fidelity factor for face to face and 91.2% for side by side.
[37]	Presents ring antenna biosensor design to detect glucose.	Used simple and cost-efficient structure of split-ring resonator.	7.3% highest error rate and 17.5 MHz frequency shift in 15 min with a sensitivity of 0.107 MHz/mg mL${}^{-1}$.
[38]	Implements a metamaterial antenna array for sensing and imaging applications.	Provides weak signal detection and reduces distortion.	Meets 11 dBi average radiation gain, 18% efficiency improvement, 30 dB high average isolation.
[39]	Uses continuous waves with an antenna array to remotely monitor the human respiratory rate system.	The ability to image the respiratory system and find an accurate rate.	Obtain 0.05 m average imaging error with a 30 ms error in estimating the RI.
[40]	Provides a photoconductive photomixer array continuous THz wave for biomedical imaging.	Few THz micro-watt power is achievable from the photoconductive array at 1 THz.	A possibility of adjusting the angle between two rays with 30${}^{\circ }$ directionality.
[41]	Provides surface plasmon resonance that can be used in tumor detection, imaging, and sensing applications.	Improvements in dye molecule absorption, lifetime, and quantum efficiency with two nanoparticles.	Shows Si/SiO_2_/Ag near-field excitation and factor improvement is higher than Au/SiO_2_/Au nanoparticles.
[42]	Provides a wireless and non- battery trimodal neural interface system-on-chip.	Supports 16 channels of neural recording together with channel optical stimulation and 8-channel electrical stimulation.	An analog front-end of 55–70 dB with low/high cutoff frequencies of 1–100 Hz/10 kHz.

**Table 3:** A Synopsis of Current Methods and Approaches Employed in Remote Health Monitoring.

Ref.	Scope	Contribution	Specifications
		**Channel Characteristics**	
[43]	Provides a design of the human body communication channel using the impulse response.	Investigates human body channels and shows how to improve the accuracy further to send reliable data.	The impulse response random variables are uniformly distributed and reflecting the dependency enhances the accuracy.
[44]	Provides analysis for in/off body ultra-wideband communication channels.	The needed transmission power is analyzed using three techniques to improve the data rates and transmitted power.	10× improvements in practical schemes are obtained while maintaining the capacity and minimizing the power.
[45]	Investigates dual analyte channel using biosensor of surface plasmon resonance.	Minimizes the surface roughness and the metal deposition on analyte channels.	Meets the requirements with 186,000 nm/RIU wavelength sensitivity, 2792.97 ${\mathrm{RIU}}^{-1}$ amplitude sensitivity, and 0.0204 ${\mathrm{nm}}^{-1}$ detection accuracy.
[46]	Investigates interbody channels at both optical and THz bands for Invivo wireless nanosensor networks.	Finds the path loss by including human tissue molecular absorption and small and large-scale scattering.	The particle size parameter was bigger than one with scattering efficiency near two which verifies optical principles.
[47]	Studies body antenna network propagation.	Provides power effective propagation using Norton wave mechanism at 3 GHz.	Path gain is independent of the frequency. UWB will result in dispersionless.
[48]	Analysis using integrating dual data rate receiver for Broadband-Human body communication.	Works with three methods: continuous wave, amplitude modulation, and frequency modulation.	Meets 22 dB SIR improved efficiency for AM and FM plus 10${}^{-4}$ BER and −21 dB efficiency in interference rejection.
		**WCE Advancements**	
[49]	Proposes radio frequency backscatter for high data rate deep implants.	Provides a power-efficient and efficiency improvement model for wireless capsule endoscopy.	Meets 1 and 5 Mb/s backscatter data connectivity at 13 cm depth with a moderate reader power of 250 mW and reduces the power by 20–45 mW.
[50]	Presents wireless capsule endoscopy localization investigation.	Discusses different parameters that affect the system like array sensor, organ properties, and transmitting signals.	A high effect of localization when an external antenna is used with capsule number in the GI tract and impacted large intestine using the power of transmitter probability distribution.
		**Sensing and Imaging**	
[51]	Overviews different techniques to solve the spatial resolution problem for optical brain imaging.	Improvements in the spatial resolution which is used for brain image applications and compared to another method.	Investigates OBI systems bottleneck and challenges.
[52]	Overviews different physical sensing methods for wearable health-monitoring devices.	Provides up-to-date literature review that uses new methodology.	Shows printed interface circuits used with wearable sensors to monitor biometric parameters.
[53]	Studies the disturbed signal transmission for mm-wave glucose sensing application.	Proposes absorbers that are used around the tissue to reduce the effect of multipath transmission and increase signal sensitivity.	5 wt% glucose in DI water was obtained for two acrylic containers and it showed that the absorbers increase the amplitude of $\Delta S_{21}$.
		**Channel Characteristics**	
[54]	Investigates near-filed THz imaging on different biological samples using three probes horn antennas.	Overcomes the spatial resolution problem of far-field imaging methods.	Meets LD = 445 μm metal probe with 0.8375 THz, LD = 75 μm quartz probes with 0.8024–0.8424 THz, LD = 230 μm telfon probes with 1.0422 THz.
[55]	Proposes ultrashort pulse microwave-induced thermoacoustic imaging technique with aperture antenna for clinical breast and tumor screening.	Solves large-field imaging issues.	Provides 40 cm × 27 cm microwave radiation, 14 cm × 14 cm uniform imaging, a 7 cm depth of imaging, and a resolution of 290 μm.

## Nomenclature

**Abbreviation**	**Definition**
AM	Amplitude Modulated
AS	Amplitude Sensitivity
BAN	Body Antenna Network
BER	Bit Error Rate
BW	Bandwidth
B5G	Beyond Fifth Generation of Mobile Networks
CP	Circularly Polarized
CW	Continuous Wave
DMA	Dynamic Metasurface Antennas
EM	Electromagnetic
FEM	Finite Element Method
5G	Fifth Generation of Mobile Networks
FM	Frequency Modulated
GI	Gastroin-testinal
GMS	Gradient Metasurface
GST	$Ge_{2}Sb_{2}Te_{5}$ Material
HBC	Human Body Communication
IoMT-based WBAN	Internet of Medical Things-based Wireless Body Area Network
IR	Impulse radio
ISM	Industrial, Scientific, and Medical
LoS	Line of Sight
LP	Linear Polarization
LWA	Leaky-wave Antenna
mm-Wave	Millimeter Wave
MRI	Magnetic Resonance Imaging
NIR	Near Infrared
NLoS	Non-Line of Sight
NP	Nanoparticle
OBI	Optical Brain Imaging
OFDM	Orthogonal Frequency Division Multiplexing
OOK	On-off Keying
PCF	Photonic Crystal Fiber
PCR	Polarization conversion rate
PDMS	Polydimethylsiloxane
PLL	Phase-locked Loops
PPM	Pulse-position Modulation
QPSK	Quadrature Phase Shift Keying
RF	Radio Frequency
RI	Refractive Index
RMS	Root Mean Square
RS	Reed Solomon
SNR	Signal-to-Noise Ratio
SoC	Interface System-on-chip
SP	Surface Plasmon
SPR	Surface Plasmon Resonance
SSPs	Spoof Surface Plasmon Polaritons
TDM	Time Division Multiplexing
THz	Terahertz
ToA	Time of Arrival
UWB	Ultra Wideband
WBAN	Wireless Body Antenna Network
WCE	Wireless Capsule Endoscope
WGM	Whispering-gallery-mode
WLAN	Wireless Local Area Network
WMTS	Wireless Medical Telemetry Service
WS	Wavelength Sensitivity
